# 한국 가임기 여성의 COVID-19 백신 접종이 월경 주기 변화에 미치는 요인: 탐색적 상관성 연구

**DOI:** 10.4069/whn.2025.12.09

**Published:** 2025-12-31

**Authors:** Sunhee Lee, Kyung-Eui Bae, Hyun-Jung Park

**Affiliations:** 1 Department of Nursing, Baekseok Cultural University, Cheonan, Korea; 1 백석문화대학교 간호학과; 2 Department of Nursing, Dongseo University, Busan, Korea; 2 동서대학교 간호학과; 3 Department of Nursing, Pyeongtaek University, Pyeongtaek, Korea; 3 평택대학교 간호학과

**Keywords:** COVID-19 vaccines, Menstrual cycle, Reproductive health, Uterine hemorrhage, Korea

## Abstract

**Purpose:**

This study aimed to examine the impact of coronavirus disease 2019 (COVID-19) vaccination on menstrual cycle changes among Korean women of reproductive age.

**Methods:**

This exploratory correlational study used data from an online, self-administered questionnaire obtained from the Korea Social Science Data Center database. The survey assessed menstrual cycle changes following COVID-19 vaccination among Korean women of reproductive age (15–49 years) who were menstruating regularly and had a history of COVID-19 vaccination. Data were collected between February and March 2023. A total of 227 participants who completed the online questionnaire were included in the analysis.

**Results:**

The risk of menstrual cycle changes was higher among older women (reference group: 40–49 years; 15–19 years: odds ratio [OR], 0.25 [*p*=.006]; 30–39 years: OR, 0.33 [*p*<.001]), women with obesity (reference group: body mass index [BMI] >25 kg/m^2^; BMI <18.5 kg/m^2^: OR, 0.03 [*p*=.030]; BMI 18.5–22.9 kg/m^2^: OR, 0.002 [*p*=.002]), and unmarried women (OR, 3.02). Increased risk was also observed among participants with a history of COVID-19 infection (OR, 3.46; *p*=.015), those who received the second or lower dose of the vaccine, and those reporting excessive menstrual flow (OR, 2.07; *p*=.015), intermenstrual symptoms (OR, 2.72; *p*=.037), or irregular menstruation (OR; 17.26, *p*<.001).

**Conclusion:**

Menstrual cycle changes following COVID-19 vaccination appear to be influenced by multiple factors, particularly the interaction between a history of irregular menstruation and environmental conditions. Further longitudinal studies and tailored management strategies for specific subgroups, including unmarried and obese women, are needed to support women’s health and quality of life.

## Introduction

코로나바이러스감염증-19 (coronavirus disease 2019, 이하 COVID-19) 팬데믹은 약 3년간 전 세계 모든 사람의 삶에 신체적 건강뿐만 아니라 심리적, 경제적, 정치적 영향을 미쳤다. 바이러스의 급속한 확산으로 인한 감염과 입증된 치료법의 부족으로 인해 전 세계에 파급되는 영향을 해소하기 위해 미국 식품의약국(Food and Drug Administration)은 2020년 12월 11일과 18일에 각각 Pfizer/BioNTech와 Moderna 백신에 대한 긴급 사용을 승인하였다[1]. 많은 사람이 이처럼 신속하게 승인된 백신을 접종받았는데, 모든 의약품과 마찬가지로 COVID-19 백신도 부작용을 일으킬 수 있으나 새롭게 승인된 이 백신들은 긴급 사용 승인을 받으면서, 정식 임상시험을 완료하지 않아 충분한 과학적 연구 부족과 알려지지 않은 장기적인 부작용 등에 대한 큰 우려가 제기되었다. 승인된 COVID-19 백신을 접종한 사람들은 발열, 오한, 두통, 피로, 팔 통증과 같은 단기적인 경미한 증상부터 아나필락시스와 관련된 입술, 얼굴, 또는 혀 부종 및 심근경색과 혈전증과 같은 심각한 부작용까지 다양한 백신 관련 부작용을 보고하였다[2-5]. 또한, 가임기 여성들은 월경 주기의 불규칙성과 임신 지연을 보고하여 가임기 성인 여성의 생식 건강에 영향을 미칠 수 있다는 우려가 제기되었다[6,7].

여성들의 월경 관련 보고를 살펴보면, 미국 백신 부작용 보고 시스템(Vaccine Adverse Event Reporting System, VAERS)에서 2022년 4월까지 COVID-19 백신 접종 후 월경과 관련된 발표를 검색한 연구에서[8], 11,000명 이상이 과다 출혈, 불규칙하거나 지연된 월경, 희발월경, 무월경 등 월경과 관련하여 자가 보고한 증상이 있었다고 하였다. 또한, 영국 의약품 및 의료 규제 기관(Medicines & Healthcare Products Regulatory Agency, MHRA)의 옐로카드 감시 제도는 5만 건 이상이 평소보다 많은 월경량, 월경 지연, 예상치 못한 출혈 등 월경 장애 신고를 접수하였다[9]. 우리나라 백신안정성연구센터(VSRC, Vaccine Safety Research Center)의 2023년 3월 발표 자료에 의하면, 백신 접종 여성 10만명 당 20명이 이상 자궁출혈을 보고하였고 이 중 79%가 의료기관을 방문하여 치료를 받았다[10-12]. 또한, 우리나라 COVID-19 백신 안정성위원회에서는 2022년 8월에 COVID-19 백신 접종을 받은 16–64세 여성을 대상으로 COVID-19 백신 접종과 빈발월경 및 출혈 관련 이상 자궁출혈 등 월경 주기 간의 연관성이 있음을 발표하였다[11].

월경 주기의 불규칙성은 대사증후군, 이상지질혈증 및 난임 발병 위험을 증가시키고[7], 월경 주기가 불규칙하거나 긴 여성은 70세 이전에 사망할 위험이 더 높다[13]. 여성들은 COVID-19 백신 접종 후 월경 주기 및 월경량 증가, 통증, 이상 자궁출혈 등 여러 가지 월경 이상 증상 및 월경 주기 장애를 경험하고[11-12,14-18] 일부 여성에서는 임신 지연을 보였다[6-7]. 월경 주기는 건강과 생식력의 명백한 신호이며 여성의 전반적인 건강 상태를 반영하는데, 월경 주기의 불규칙성에 대한 결과들은 여성의 생식 건강, 특히 가임기 여성의 생식 건강에 위협을 줄 수 있다[8,15-16].

월경 주기와 COVID-19 백신과의 연관성에 대한 연구는 상반된 결과를 보인다. COVID-19 백신 종류와 백신 접종 후 월경 주기의 규칙성, 지속 기간의 변화, 통증 강도의 변화 사이에는 유의미한 연관성이 있었다[8,12,14-17]. 그러나 COVID-19 백신 접종이 월경 시작, 월경 기간, 월경량 및 연령, 체질량지수, 흡연과 같은 인구∙사회학적 변수가 월경 주기 변화와 유의미한 연관성을 보이지 않은 결과도 있다[14]. 한편, 인간면역결핍 바이러스, B형 간염, C형 간염과 같은 일부 바이러스 감염은 생식계 합병증 및 월경 주기 변화와 관련이 있는 것으로 보고되었으며, COVID-19 바이러스 감염은 무월경, 월경 과다, 장기간 및 과다 월경, 소량 및 저월경, 월경 전 증후군(premenstrual syndrome, PMS) 관련 다양한 증상, 월경통을 유발할 수 있어[19] COVID-19 바이러스 감염도 여성의 월경 주기 변화에 영향을 미칠 수 있다는 것을 알 수 있다. 이러한 결과들은 백신 접종 후 월경 이상이 다양한 인구 집단(연령별•비만 유무•미혼 여부 등)에서 다인성 원인(여성의 기저 월경 이상, 스트레스, 감염력 등)과 상호작용을 보이면서 발생하고 있다[6,8,12,14-17]. 특히 월경 주기가 불규칙했던 여성, 비만 여성, 미혼 여성, COVID-19 감염력이 있었던 여성 등이 백신 접종 후 월경 이상을 경험할 위험이 상대적으로 높다[19].

가임기 여성들 사이에서 백신 접종 후 월경 주기 장애를 유발하고 임신 가능성에 영향을 미칠 수 있다는 우려는 꾸준히 제기되고 있으므로[6-7,17] 한국 가임기 여성을 대상으로 COVID-19 백신과 월경 주기 변화와의 연관성을 확인해 볼 필요가 있다. 국내에서 한국 여성을 대상으로 한 COVID-19 백신 접종과 월경 관련 조사에서는 COVID-19 백신 접종 후 월경 주기, 월경 기간, 월경량, 이상 자궁출혈의 유병률이 통계적으로 유의미한 차이가 있었다[12]. 그러나 가임기 여성을 대상으로 COVID-19 백신 접종과 월경 주기 또는 월경 이상과 관련된 국내 간호학 분야의 관련 연구는 매우 제한적이다. 이에 한국 가임기 여성의 COVID-19 백신 접종이 COVID-19 바이러스 감염 이력을 포함한 인구∙사회학적 특성, 월경 이상 증상, 백신 접종 횟수와 백신 종류에 따라 월경 주기 변화에 미치는 영향을 조사하고자 한다. 이는 여성 생식기 건강에 대한 보건 정책 수립의 근거 자료 및 간호실무에서 백신 접종 전후 여성 건강 상담을 위한 기초 자료를 제공할 뿐만 아니라 COVID-19 백신 접종에 따른 월경 주기 변화에 대한 국내 간호학 분야에 기초 자료를 제공한다는 의의가 있다.

구체적인 연구 목적은 다음과 같다.

첫째, 연구 대상자의 COVID-19 백신 접종 후 월경 주기 변화에 따른 인구∙사회학적 요인을 파악한다.

둘째, 연구 대상자의 COVID-19 백신 접종 후 월경 주기 변화에 따른 월경 이상 증상을 파악한다.

셋째, 연구 대상자의 COVID-19 백신 접종 후 월경 주기 변화에 따른 백신 접종 횟수, 백신 종류, 월경 이상 증상 관리를 파악한다.

넷째, COVID-19 백신 접종 후 월경 주기 변화에 영향을 미치는 요인을 파악한다.

## Methods

**Ethics statement:** This study was approved by the Institutional Review Board of Gimcheon University (No. IRB- GU-202212-HRa-12-02-P). Informed consent was obtained from the participants

### 연구 설계

본 연구는 가임기 여성의 COVID-19 백신 접종이 월경 주기 변화에 영향을 미치는 요인을 파악하기 위한 탐색적 상관성 연구이다. 본 연구는 STROBE 지침(https://www.strobe-statement.org/)을 준수하여 기술하였다.

### 연구 대상

본 연구의 대상자는 가임기 여성(15–49세) [10]으로 평상시 규칙성을 가지고 월경을 하고 있으면서 1회 이상의 COVID-19 백신 접종 이력이 있는 한국 여성이었다. COVID-19 백신 미접종자, COVID-19 백신 접종을 하였으나 평상시 월경이 불규칙한 여성, 만 15세 미만이나 폐경기 무렵 여성으로 생리적으로 불규칙한 월경의 가능성이 있는 여성, 생식기계 관련 질환, 갑상선 질환, 만성질환자는 제외하였다.

본 연구의 표본 크기 산출에는 G*Power ver. 3.1.9.2 (Heinrich Heine University Düsseldorf, Düsseldorf, Germany) 프로그램을 사용하였다. 로지스틱 회귀분석(logistic regression)에서 최소 표본 크기를 구하기 위해서는 효과 크기(odds ratio, OR)와 귀무가설 하 사건의 비율 p₁=P(Y=1|H₀)의 설정이 필요하다. 사건의 비율은 본 연구의 종속변수인 COVID-19 백신 접종 후 월경 주기 변화 발생 여부에 관한 기존 문헌의 발생률을 근거로 산정하였다. 가임기 여성을 대상으로 한 선행 연구에서 백신 접종 후 월경 주기 변화는 약 20%에서 40% 수준으로 보고된 바 있다. Edelman 등[16]은 백신 접종군에서 대조군 대비 월경 주기 길이가 유의하게 증가하였으며 전체 변화율은 약 38%였다고 보고하였고, Li 등[1]은 COVID-19 감염 및 면역 반응과 관련된 호르몬 변화가 월경 이상과 연관됨을 보고하였다. 이를 종합하여 본 연구에서는 귀무가설 하 사건의 비율을 p₁=0.30으로 설정하였다. 효과 크기(OR)는 월경 변화에 영향을 미치는 생리적•면역학적 요인들이 관련 사건 발생 위험을 약 1.3–1.8배 증가시키는 기존 연구 결과[1,2]를 근거로 하여, 본 연구 맥락에서 임상적으로 의미 있는 최소 차이를 반영한 OR을 1.5로 설정하였다. 검정유형은 양측검정, 유의수준 α는 0.05, 검정력 1–β=0.80으로 설정하였다. 위의 가정(p₁=0.30; OR, 1.5)에 따라 G*Power 3.1.9.2에서 로지스틱 회귀분석(z-test)을 이용하여 최소 표본 수를 산출한 결과, 필요 표본 크기는 약 200명으로 나타났다. 본 연구에서는 자가보고식 설문조사에서의 약 20%의 탈락률[20]을 고려하여 240명을 대상으로 설문지를 배포한 결과, 235명(응답률 97.9%)이 응답하였다. 온라인 설문에 응답한 총 235명 중 COVID-19 백신 미접종자 1명, 산부인과 질환 환자 4명, 갑상선질환 환자 2명, 만성질환자 1명 등을 제외하고(탈락률 3.4%) 총 227명을 분석에 이용하였다.

### 측정 도구

본 연구의 도구는 Muhaidat 등[21]이 개발한 설문지를 이메일을 통해 도구 허락 승인을 받은 후 본 연구자가 우리나라 실정에 맞게 일부 수정 보완한 연구 도구를 사용하였다. 원 설문지 구성은 6개 영역, 28개의 상세한 자기 보고식으로 구성되어 있으며, 한국의 지역적, 보건의료적 특성을 고려하여 여성건강간호학 전공 박사 학위자 3인의 검토 과정을 거쳐 타당성을 확보하였다. 타당성이 확보된 도구를 번역문의 언어적 오류와 전문 용어 사용에 따른 문장 이해 정도를 점검하기 위해, 서울과 경기도 10명, 충청도 10명, 경상도 10명 등 총 30명을 대상으로 점검 과정을 거쳐 문항을 질문 의도가 명확한 문장으로 수정하고, 의미가 중복되는 문항은 통합하고, 범위가 중복되는 답가지는 답가지를 추가하여 최종 6개 항목, 32개 문항으로 구성하였다. 최종 연구에 사용된 각 항목별 측정 도구의 구성은 다음과 같다.

#### 월경 관련 특성

월경 주기 및 월경 기간, 전년도 피임 방법, 임신 및 폐경 상태 등 총 6문항으로 구성하였다.

#### COVID-19 감염 특성

COVID-19 감염 여부와 COVID-19 백신의 일반적 부작용에 대한 3문항으로 구성하였다.

#### COVID-19 백신 접종

COVID-19 백신 관련 항목은 접종 횟수, 접종한 백신의 종류, COVID-19 백신 부작용의 심각성에 대한 총 3문항을 구성하고, 응답 문항에 4차 추가 접종 여부를 추가하였다.

#### COVID-19 백신 접종 후 월경 이상

COVID-19 예방 접종 후 월경 이상에 대한 질문은 백신 접종 후 새로운 증상, 생리 이상 지속 기간, 백신 접종 횟수, 증상 대처 방법, 삶의 질에 부정적 영향, 증상의 심각성 등 6문항으로 구성하였다.

#### COVID-19 백신 접종 후 월경 주기 변화

백신 접종 후 월경 주기 변화에 대한 측정은 Muhaidat 등[21]의 연구에서 COVID-19 백신 접종 전의 월경 주기(예, 28일)와 월경 기간(예, 5일)과 접종 후 월경 주기(예, 28일)와 월경 기간(예, 5일)에 대한 개방형 질문으로 원 도구와 동일하게 구성하였다. 백신 접종 전 월경 주기(예, 28일)에서 월경 주기 변동(예, 20일, 30일 등)으로 응답한 경우 월경 불순, 8일 이상의 월경 기간을 응답한 경우는 월경 기간 연장, 월경과 월경 사이에 출혈이 발생을 응답한 경우 월경 간 출혈, 월경량이 많아진 경우는 월경 과다, 백신 접종 전 PMS 증상이 없었으나 접종 후 나타났거나 증상이 심하게 된 경우 PMS의 악화로 정의하였다..

### 일반적 특성

연령, 거주지역, 키와 몸무게, 최종학력, 혼인상태, 자녀 수, 기존 의학적 진단 질환 및 흡연, 음주에 대해 총 10문항으로 구성하였다.

### 자료 수집 기간 및 방법

본 연구는 온라인 설문을 통해 자료를 수집하였고, 한국 시간 기준으로 2023년 2월 24일 오전 10시에 시작하여 3월 9일 오후 7시에 마감하였다. 한국사회과학 데이터 센터(Korean Social Science Data Center) 데이터베이스의 온라인 설문 시스템을 통한 자기 기입식 조사로, 연구의 설명문을 읽고 동의서에 동의한 자를 연구 대상자로 하였다. 연구자는 가임기 여성이 많이 참여하는 사회관계망 서비스를 통해 연구 목적, 연구 대상자 조건 및 제외 조건을 작성한 모집 공고문을 배포하고, 연구 목적에 적합한 대상자가 연구에 참여할 의사가 있는 경우 설문 링크에 접속하도록 하였다. 또한, 설문에 응답한 대상자에게 연구 대상자 조건에 해당하는 지인이 있을 경우 설문 링크를 공유해 주도록 하는 눈덩이 표집 방법으로 자료를 수집하였다.

연구 설명문에는 연구의 목적과 온라인 설문 방법에 대해 설명하였고, 연구에서 취득한 개인정보나 연구 설문 내용은 연구 목적으로만 사용하며 익명성을 보장함을 안내하였다. 또한 설문 조사를 중단하여 연구 참여를 중단할 수 있으며 어떠한 불이익도 발생하지 않음을 설명하였다. 설문을 완료한 참여자에게는 모바일 상품권(2,000원 상당)이 발송되며 원하는 경우에만 핸드폰 번호를 입력하도록 하였고 수집된 연락처는 모바일 상품권 발송 직후 폐기하였다.

### 자료 분석

본 연구의 자료 분석은 IBM SPSS for Windows, ver. 29.0 (IBM Corp., Armonk, NY, USA)를 이용하여 분석하였다. 연구 대상자의 인구∙사회학적 특성으로 조사된 변수에 따라 COVID-19 관련 특성, COVID-19 백신 접종 후 인구∙사회학적 특성 및 COVID-19 감염 여부, 백신 접종 수, 접종 후 월경 이상 증상은 교차분석으로 분석하였고, 이상 증상이 나타난 접종 횟수, 백신 접종 종류, 이상 증상이 나타난 후 대처방법 등은 다중응답 교차분석으로 분석하였다. 가임기 여성의 COVID-19 백신 접종 후 월경 주기의 변화에 관련성을 보이는 요인을 확인하기 다변량 로지스틱 회귀분석을 시행하여 교차비와 95% 신뢰구간, *p*값을 이용하여 검정하였고, 통계적 유의수준은 *p*<.05로 분석하였다.

## Results

### COVID-19 백신 접종 후 월경 주기 변화에 따른 일반적 특성

COVID-19 백신 접종 후 월경 주기 변화 유무에 따른 일반적 특성을 파악해 본 결과, 연령, 체질량지수, 혼인 상태, 음주 여부, 과거 COVID-19 감염력, 백신 접종 횟수에서 통계적으로 유의한 차이가 나타났다. 월경 주기 변화가 있는 집단(111명)에서는 40–49세가 31명(13.7%)으로 변화가 없는 집단(116명)의 13명(5.7%)에 비해 높은 비율을 보였으며(*χ*^2^=9.32, *p*=.010), 비만(≥25 kg/m^2^)이 변화 있음 집단에서 15명(6.6%)으로 변화 없음 집단의 2명(0.9%)보다 현저히 높게 나타났다(*χ*^2^=28.68, *p*<.001). 또한 비혼자의 비율이 변화 있음 집단에서 80명(35.2%)으로 변화 없음 집단(65명, 28.6%)에 비해 높았으며(*χ*^2^=6.32, *p*=.012), 음주를 하는 경우에도 변화 있음 집단 67명(29.5%)이 변화 없음 집단 86명(37.9%)과 차이를 보였고, 비음주자는 변화 있음 집단 44명(19.4%), 변화 없음 집단 30명(13.2%)이었다(*χ*^2^=4.90, *p*=.023). COVID-19 감염 이력이 있는 경우 변화 있음 집단 99명(43.6%), 변화 없음 집단 78명(34.4%)으로 차이가 있었으며(*p*<.001), 2차 이하 접종자 또한 변화 있음 집단 70명(30.8%), 변화 없음 집단 57명(25.1%)에 비해 상대적으로 높았다(*χ*^2^=4.46, *p*=.035). 한편, 흡연 여부, 자녀 유무, 거주 지역에 따라서는 유의한 차이가 관찰되지 않았다(Table 1).

### COVID-19 백신 접종 후 월경 주기 변화에 따른 월경 이상 증상

COVID-19 백신 접종 후 월경 주기 변화 유무에 따른 월경 이상 증상 발생을 분석한 결과, 월경 이상이 있는 집단(111명)에서 불규칙 월경(59명, 26.0%)이 변화 없음 집단(116명)의 10명(4.4%)보다 유의하게 높게 나타났으며, 규칙적인 월경(21–35일) 비율은 변화 없음 집단이 106명(46.7%)으로 변화 있음 집단의 52명(22.9%)보다 높았다(*χ*^2^=53.17, *p*<.001). 과다월경은 변화 없음 집단 13명(5.7%), 변화 있음 집단 42명(10.1%)으로, 변화 있음 집단에서의 발생 빈도가 통계적으로 유의하게 높았다(*χ*^2^=21.91, *p*<.001). 간헐적 출혈의 경우 변화 있음 집단이 31명(13.7%)으로 변화 없음 집단(16명, 7.0%)에 비해 더 많이 나타나 유의한 차이를 보였다(*χ*^2^=6.90, *p*=.009). 월경 기간의 연장(8일 초과)은 두 집단 간 차이가 없었다(*χ*^2^=.07, *p*=.474). PMS 악화는 변화 없음 집단 23명(10.1%), 변화 있음 집단 36명(15.9%)으로 변화 있음 집단에서 더 흔하게 보고되었으며(*χ*^2^=4.66, *p*=.030), 월경 이상이 있는 경우 다양한 증상을 복합적으로 경험할 위험이 더 높음을 보여주었다(Table 2).

COVID-19 백신 접종 후 월경 주기 변화에 따른 백신 접종 횟수, 백신 종류, 월경 이상 증상 관리 방법

COVID-19 백신 접종 후 월경 주기 변화에 따른 백신 접종 횟수, 종류, 월경 이상 증상 관리 방법은 다중응답 교차분석을 실시하였다. COVID-19 백신 접종 후 월경 이상 경험에 대한 분석 결과, 전체 227명 중 111명(48.9%)이 월경 이상을 경험하였다. 증상 발현은 4차 접종 후가 94명(27.4%)으로 가장 많았고 3차 접종 87명(25.4%), 1차 접종 89명(25.9%), 2차 접종 73명(21.3%) 순으로 나타났다. 월경 변화를 경험하지 않은 군에서는 1차 접종 후 증상 발현이 34명(56.8%)으로 가장 높았고, 월경 변화를 경험한 군에서는 4차 접종 후가 61명(17.8%)으로 가장 높았다. 백신 유형별로는 Pfizer-BioNTech가 399명(43.9%)으로 가장 많이 접종되었고, Moderna 78명(8.6%), AstraZeneca 74명(8.1%) 순이었다. 월경 이상 관리 방법으로는 친척이나 지인과 상담하는 경우가 84명(51.2%)으로 가장 많았고, 온라인 증상 검색 35명(21.3%), 일차 의료진 상담 24명(14.6%) 순이었다. 월경 변화가 있는 군에서는 온라인 검색 25명(15.2%), 일차 의료진 상담 20명(12.2%), 자가치료 14명(8.5%)의 순서로 대처하였으며, 산부인과 전문의 진료는 월경 변화가 있는 군에서만 4명(2.4%)이 받았다(Table 3).

### COVID-19 백신 접종 후 월경 주기 변화에 영향을 미치는 요인

COVID-19 백신 접종 후 월경 주기 변화 영향 요인에 대한 다변량 로지스틱 회귀분석 결과에서, 연령, 체질량지수, 혼인 상태, COVID-19 감염력, 이상 증상이 나타나기 시작한 백신 접종 횟수, 간헐적 출혈, 과다월경, 월경의 규칙성을 변수로 투입하였을 때 COVID-19 백신 접종 후 월경 주기 변화에 영향을 미치는 여러 요인이 확인되었다. 연령별로 살펴보면 40–49세를 기준군으로 비교하였을 때 15–19세 여성이 월경 변화의 위험이 약 75% 낮았으며(OR, 0.25; *p*=.006), 30–39세 역시 약 66% 낮은 위험을 보여 유의미한 차이를 보였다(OR, 0.33; *p*<.001). 체질량지수 분석에서는 기준 군인 비만군에 비해 저체중(<18.5 kg/m^2^)의 경우 월경 주기 변화 위험이 97% 이상 낮았고(OR, 0.03; *p*=.030), 정상 체중(18.5–22.9 kg/m^2^)의 경우에도 약 89% 이상 낮은 위험을 나타내었다(OR, 0.002; *p*=.002). 이는 비만한 여성보다 저체중 또는 정상 체중 여성에서 백신 후 월경 변화가 발생할 가능성이 낮음을 시사한다. 비혼 여성은 기혼 여성보다 월경 주기의 변화 발생 위험이 약 3배 더 높았으며(OR, 3.02; *p*=.015), COVID-19 감염 이력이 없는 여성 역시 감염 이력이 있는 여성보다 약 3.5배 더 높은 위험을 보였다(OR, 3.46; *p*=.015). 백신 접종 수 2회 이하인 경우가 3회 이상인 경우보다 월경 주기 변화 위험이 약 2배 가까이 증가하였다(OR, 2.07; *p*=.015). 부정출혈이 있는 경우 월경 변화 위험은 약 2.7배 더 높았고(OR, 2.72; *p*=.037), 과다월경이 있는 경우에는 5배 이상 높았다(OR, 5.37; *p*<.001). 특히, 월경 주기가 불규칙한 여성은 규칙적인 여성에 비해 월경 변화 위험이 무려 17배 이상 증가하는 것으로 나타났다(OR, 17.26; *p*<.001) (Table 4).

## Discussion

본 연구는 한국 가임기 여성에서 COVID-19 백신 접종 이후 보고된 월경 주기 변화의 양상과 관련 요인을 인구∙사회학적 특성, 월경 이상 증상, 백신 관련 변수 및 다변량 영향요인 분석을 통해 포괄적으로 확인하고자 하였다. 연구 대상자의 약 절반에서 접종 후 월경 변화가 보고되었으며, 연령, 체질량지수, 혼인 상태, COVID-19 감염력, 접종 횟수, 월경 불규칙성, 과다월경, 간헐적 출혈 등이 유의한 관련 요인으로 나타났다. 이러한 결과는 COVID-19 백신이 가임기 여성의 월경 양상에 영향을 미칠 수 있다는 최근 국내•외 연구들과 맥락을 같이 하면서도[6,12,16,17,20,22-24] 한국 가임기 여성 집단의 특성을 반영한 근거를 제시한다는 점에서 의의가 있다.

연령과 체질량지수, 혼인 상태, 감염력, 음주 여부 등 인구∙사회학적 특성은 COVID-19 백신 접종 후 월경 주기 변화와 유의한 차이를 보였다. 연령이 증가할수록 월경 변화 보고가 증가하였고, 특히 40–49세에서 위험이 상대적으로 높게 나타났다. 여러 국가에서 수행된 연구들 역시 연령대에 따라 월경 변화 보고 양상이 다르게 나타났으나, 특정 연령대에 집중된 변화보다 각 연구의 표본 구성과 접종 시기가 다름에 따른 이질성이 강조된 바 있다[6,17,20,22-24]. 따라서 본 연구에서 관찰된 고연령층의 높은 변화 보고는, 연령 증가에 따른 기저 월경 변동성, 만성질환 및 약물 복용, 건강에 대한 민감한 인식 등이 복합적으로 작용했을 가능성을 배제할 수 없으나, 이러한 요인을 직접 측정하지 못했다는 점에서 인과적 해석보다는 고연령층이 접종 이후 변화를 보다 뚜렷하게 인지했을 가능성이 있다는 수준으로 이해하는 것이 타당하다. 체질량지수는 비만 여성에서 월경 주기 변화 위험이 높게 나타난 반면, 저체중 및 정상 체중 여성은 위험이 유의하게 낮아, 비만과 월경 불규칙성 및 대사이상 간 관련성을 보고한 선행 연구[7,13]와 맥락을 같이 한다. 아시아•태평양 지역의 비만 기준[25]을 적용한 본 연구 결과는 아시아계 여성에서 비만이 백신 후 월경 변화를 포함한 생식 건강 취약성과 관련될 수 있음을 시사한다. 혼인 상태의 경우 비혼 여성이 기혼 여성보다 월경 변화 가능성이 높았는데, 이는 COVID-19 팬데믹 기간 동안 여성의 재생산 건강에 영향을 미친 심리∙사회적 스트레스, 직장•가사 부담, 건강 행태 변화가 월경 증상에 영향을 줄 수 있다는 보고와 부분적으로 연결된다[15]. 다만 본 연구에서는 스트레스, 수면, 직무 특성 등의 요인을 수집하지 않았으므로, 혼인 상태 자체를 원인으로 보기보다는 혼인 상태가 월경 주기 변화와 관련된 다양한 생활 및 건강 관련 맥락을 간접적으로 반영했을 가능성이 있으며, 향후 연구에서 이러한 맥락 변인들에 대해 좀 더 측정•통제하여 재검증할 필요가 있다.

월경 이상 증상과 관련하여, 월경 주기 변화가 있는 여성은 불규칙 월경, 과다월경, 간헐적 출혈, PMS 악화를 유의하게 더 많이 경험하였다. 특히 불규칙 월경은 다변량 분석에서 가장 높은 OR을 보여, 기저 월경 패턴의 불규칙성이 백신 이후 변화를 민감하게 반영할 수 있는 핵심 요인임을 시사한다. 월경 주기가 불규칙하거나 과다출혈을 보이는 여성에서 대사질환 및 내분비 이상 위험이 증가한다는 선행 근거[7,13]를 고려하면, 이러한 집단에서 백신 이후 발생하는 비교적 작은 변동도 더 쉽게 ‘변화’로 인지되고 보고되었을 가능성이 있다. 과다월경과 간헐적 출혈 역시 월경 변화 경험과 밀접하게 연관된 것으로 나타났으며, 이는 VAERS와 MHRA의 Yellow Card, 국내 백신 안전성 평가 자료 등 여러 감시 체계에서 COVID-19 백신 이후 보고된 월경 관련 이상 유형과 유사하다[8-11]. 미국, 사우디아라비아, 터키, 중동 및 북아프리카 지역, 이라크 등에서 수행된 관찰 연구에서도 출혈량 증가, 주기 변화, 간헐적 출혈, 무월경 등이 반복적으로 보고되고 있어[6,7,16,17,19,21-24,26], 본 연구 결과가 이러한 국제적 보고와 방향성을 같이 한다고 볼 수 있다. 반면 월경 기간의 연장은 두 군 간 유의한 차이가 없었으며, PMS 악화는 이변량 분석에서는 차이가 있었으나 다변량 분석에서는 독립적 영향 요인으로 남지 않았다. 이는 월경 양•주기 패턴 변화가 백신 후 반응의 보다 직접적인 지표로 작용하는 반면, PMS는 팬데믹으로 인한 스트레스•불안과 같은 광범위한 심리∙사회적 요인의 영향을 함께 반영하는 증상 군임을 시사한다[15,21]. 따라서 PMS 변화는 백신 자체의 효과라기보다는, 팬데믹과 백신 접종이라는 복합적 상황이 여성의 주관적 월경 경험에 미치는 영향을 함께 보여주는 지표로 해석하는 것이 적절하다.

백신 관련 변수 측면에서, 접종 횟수와 백신 종류는 월경 주기 변화와 일정 부분 관련성을 보였다. 증상 발현 시점은 4차 접종 후가 가장 많았으나, 다변량 분석에서는 2회 이하 접종자의 월경 변화 위험이 3회 이상 접종자보다 높게 나타났다. 이는 접종 횟수의 증가가 선형적으로 위험을 높인다고 보기보다는, 각 접종 시점에서의 백신 공급 상황, 대상자 선정 기준, 개인의 건강상태 및 팬데믹 국면 등에 따라 월경 변화가 다르게 인지•보고되었을 가능성을 시사한다. 실제로 다른 국가의 연구에서도 접종 횟수와 월경 변화 간 관계는 일관되게 보고되지 않았으며[7,17,19,21-23,26], 일부 연구에서는 1•2차 접종 직후에 변화가 집중되거나[17,20], 부스터 접종 이후 보고가 증가하는 패턴[7,21,23]이 관찰되었다. 따라서 접종 횟수와 월경 변화의 관계는 횟수 자체보다는 접종 시기의 맥락과 결합하여 해석할 필요가 있다. 백신 종류와 관련해서 본 연구에서는 Pfizer-BioNTech 접종자가 가장 많았는데, 이는 국내 도입•접종 비율과 백신 정책을 반영한 결과로서[2,3,5,27], 특정 백신 플랫폼이 다른 플랫폼에 비해 월경 변화 위험이 더 높다고 단정하기에는 근거가 부족하다. VAERS, MHRA의 Yellow Card 및 국내 백신 안전성 연구에서도 다양한 종류의 COVID-19 백신에서 월경 관련 이상이 보고되고 있으며[8-11], 이는 백신 플랫폼 차이뿐 아니라, 보고 체계, 인지도, 접종 대상자 특성 등이 함께 영향을 미쳤음을 시사한다[10,11,27].

월경 이상 증상 관리 방법은 연구의 핵심 문제와 직접적인 인과 구조를 설명하는 변수는 아니지만, 여성들이 월경 변화를 어떻게 인지하고 대응하는지를 보여주는 중요한 맥락적 정보로 해석될 수 있다. 다수의 대상자가 친척•지인 상담이나 온라인 검색과 같은 비전문적 자원을 우선 활용하였고, 일차 의료진 또는 산부인과 전문의 상담은 상대적으로 적었다. 이는 월경 변화가 ‘의학적 문제’로 인식되지 않거나, 관련 정보를 제공하는 보건의료 체계에 대한 접근성•신뢰도에 한계가 있을 수 있음을 시사한다. 세계보건기구(World Health Organization)는 월경 건강을 기본적인 인권의 일부로 강조하며 월경과 관련된 정보 제공 및 의료 접근성 개선이 필요함을 제안하고 있는데[28], 본 연구의 관찰 결과는 이러한 국제적 논의와도 일정 부분 상통한다. 다만, 본 연구에서는 의료 접근성이나 의료진–환자 의사소통의 구조적 요인을 측정하지 않았으므로 이러한 해석은 탐색적 수준에 머물러야 하며, 결과를 확대하여 해석하는 데에는 신중함이 요구된다.

따라서 본 연구는 다음과 같은 몇 가지 한계점을 가진다. 첫째, 횡단적 설계와 자가보고 방식의 자료 수집에 기반하고 있어, COVID-19 백신 접종과 월경 주기 변화 간 인과관계를 명확히 규명하기 어렵다. 온라인 설문조사는 응답 탈락, 회상 및 자기선택 편향 등의 문제가 발생할 수 있으며, 이는 월경 변화의 실제 발생률과 관련 요인 추정에 영향을 미쳤을 가능성이 있다. 둘째, 편의 표본을 이용하였기 때문에 연구 결과를 모든 한국 가임기 여성에게 일반화하는 데에는 제한이 있다. 셋째, 객관적 호르몬 수치, 실제 출혈량, 디지털 월경 추적 자료 등을 포함하지 못해, 월경 변화의 생리•호르몬적 기전을 분석할 수 없었다. 넷째, COVID-19 감염력의 시기와 중증도, 정신건강, 스트레스, 수면, 약물 복용 등 잠재적 영향 변수를 충분히 통제하지 못했다.

종합적으로, 본 연구의 로지스틱 회귀분석 결과는 연령, 체질량지수, 혼인 상태, COVID-19 감염력, 접종 횟수, 불규칙 월경, 과다월경, 간헐적 출혈이 COVID-19 백신 접종 후 월경 주기 변화와 유의하게 관련된 요인임을 보여주었다. 이는 COVID-19 백신과 월경 변화 간 관계를 다룬 여러 국가의 연구에서 보고된 바와 같이, 단일 요인보다는 복수의 인구학적•임상적•월경 관련 요인이 결합하여 월경 변화를 설명한다는 점과 일치한다[6,16,17,19,21-24,26]. 특히 기저 월경 불규칙성과 과다월경은 다른 연구에서도 반복적으로 중요한 예측 요인으로 보고되어 왔으며[7,19,21,23,28], 본 연구는 이러한 경향을 한국 가임기 여성 집단에서도 확인함으로써 기저 월경 패턴을 고려한 백신 후 모니터링의 필요성을 시사한다. 또한 한국의 사회∙문화적 특성과 예방 정책을 반영한 고연령, 비만, 혼인 상태, COVID-19 감염력, 백신 접종 횟수 등에 대한 확인이 필요함은 다른 국가에서 나타난 결과와 차이가 있었다[23-26]. 국내 모바일 애플리케이션 자료를 활용한 연구에서는 백신 접종 후 월경 주기 길이의 평균 변화가 통계적으로는 유의하더라도 임상적으로는 비교적 작은 수준일 수 있음을 보여주었고[12,16], Moghadam [27]의 체계적 문헌고찰 역시 현재까지의 근거만으로는 인과관계를 단정하기 어렵다고 보고하였다. 본 연구 결과 또한 인과관계를 규정하기보다는, 월경 변화가 특정 위험 집단(고연령, 비만, 비혼, 기저 월경 이상 등)에 더 빈번히 보고될 수 있다는 점을 보여주고 있다.

이러한 맥락에서 본 연구는 COVID-19 백신 접종 전•후 상담 및 교육 시, 월경 관련 이상을 단순히 “가능한 이상반응”으로만 제시하기보다는 연령, 체질량지수, 혼인 상태, 감염력, 기저 월경 패턴 등 개인별 특성을 고려한 개별화된 설명과 모니터링 전략이 필요함을 시사한다. 특히 기존에 불규칙 월경이나 과다월경을 경험하는 여성에게는 접종 후 월경 변화 가능성을 사전에 안내하고, 변화가 지속되거나 일상생활에 지장을 줄 경우 추후 관리 및 평가를 권고하는 것이 바람직하다. 더불어, 월경 변화 경험 후 다수가 비전문적 자원을 우선 활용하였다는 점은 여성들이 월경 관련 증상을 의료진과 상의하는 데 여전히 구조적•문화적 장벽을 경험하고 있을 수 있음을 시사하므로, 월경 건강을 포함한 여성 건강 커뮤니케이션 전략과 1차 의료 영역에서의 상담 기능 강화가 중요함을 뒷받침한다[15,28].

본 연구는 인구∙사회학적 특성, 월경 이상 증상, 백신 관련 변수, 그리고 다면적 영향 요인을 동시에 고려하여 COVID-19 백신 접종 후 월경 주기 변화를 분석함으로써, 국내 가임기 여성의 월경 건강을 백신 안전성 논의의 영역에서 체계적으로 조명했다는 의의가 있다. 더불어, 다양한 지역에 거주하는 여성들을 포함한 전국 규모의 자료를 기반으로 분석을 수행하였다는 점은 연구 결과의 대표성과 해석 가능성을 강화하는 중요한 강점으로 작용하였다. 이러한 결과는 향후 국내 여성 건강 정책과 백신 안전성 평가 과정에서 월경 건강을 포함한 성별•생애주기별 지표의 중요성을 재확인하고, 여성건강 간호중재에서 더 정교한 근거 기반 의사결정을 지원하는 기초 자료로 활용될 수 있을 것이다. 향후 연구에서 제언하고자 하는 바는 전향적 코호트 설계, 모바일 애플리케이션을 활용한 실시간 월경 추적, 생물학적 표지자 측정을 포함한 다학제적 접근을 통하여, 백신과 월경 주기 변화 간 관계를 보다 정밀하고 일관되게 규명할 필요가 있으며, 다양한 연령•지역•사회문화적 배경을 가진 여성 집단을 대상으로 한 비교 연구를 통해 집단 간 차이의 원인을 탐색하는 작업도 요구된다.

## Figures and Tables

**Table 1.** General characteristics of individuals experiencing menstrual cycle changes after COVID-19 vaccination (N=227)

Characteristic	Label (range)	Post-vaccination menstrual cycle changes, n (%)		Total, n (%)	χ^2^ (*p*)
		No (n=116)	Yes (n=111)		
Region	Seoul	28 (12.3)	16 (7.0)	44 (19.4)	5.60 (.231)
	Gyeonggi-do	26 (11.5)	21 (9.3)	47 (20.7)	
	Chungcheong-do	20 (8.8)	23 (10.1)	43 (18.9)	
	Gyeongsang-do	42 (18.5)	50 (22.0)	92 (40.5)	
	Jeolla-do	0 (0)	1 (0.4)	1 (0.4)	
Age (year)	15–19	1 (0.4)	2 (0.9)	3 (1.3)	9.32. (.010)
	20–29	68 (30.3)	48 (21.1)	116 (51.1)	
	30–39	34 (15.0)	30 (13.2)	64 (28.2)	
	40–49	13 (5.7)	31 (13.7)	44 (19.4)	
Body mass index (kg/m^2^)	<18.5 (underweight)	14 (6.2)	16 (34.8)	30 (13.2)	28.68 (<.001)
	18.5–22.9 (normal)	79 (34.8)	40 (17.6)	119 (52.4)	
	23–24.9 (overweight)	21 (9.3)	40 (17.5)	25 (26.9)	
	≥25 (obesity)	2 (0.9)	15 (6.6)	17 (7.5)	
Current marital status	Unmarried	65 (28.6)	80 (35.2)	145 (63.9)	6.32 (.012)
	Married	51 (22.5)	31 (13.7)	82 (36.1)	
Child status	None	81 (35.7)	88 (38.8)	169 (74.4)	2.66 (.103)
	Exists	35 (15.4)	23 (10.1)	58 (25.6)	
Current smoking behavior	Yes	9 (4.0)	9 (4.0)	18 (7.9)	0.01 (.922)
	No	107 (47.1)	102 (44.9)	209 (92.1)	
Current drinking behavior	Yes	86 (37.9)	67 (29.5)	153 (67.4)	4.90 (.027)
	No	30 (13.2)	44 (19.4)	74 (32.6)	
Previous COVID-19 infection	Not infected	38 (16.7)	12 (55.3)	50 (22.0)	15.91 (<.001)
	Positive diagnosis	78 (34.4)	99 (43.6)	177 (78.0)	
Number of vaccinations	2nd or fewer doses	57 (25.1)	70 (30.8)	127 (55.9)	4.46 (.035)
	3rd or more doses	59 (26.0)	41 (18.1)	100 (44.1)	

COVID-19: Coronavirus disease 2019.

**Table 2.** Menstrual abnormalities in individuals experiencing menstrual cycle changes after COVID-19 vaccination (N=227)

Characteristics	Label (range)	Post-vaccination menstrual cycle changes, n (%)		Total, n (%)	χ^2^ (*p*)
		No (n=116)	Yes (n=111)		
Menstrual irregularities (day)	Regular (21–35)	106 (46.7)	52 (22.9)	158 (69.6)	53.17 (<.001)
	Irregular (<21 or >35)	10 (4.4)	59 (26.0)	69 (30.4)	
Prolonged menstrual bleeding (>8 days)	Yes	16 (7.0)	14 (6.2)	30 (13.2)	0.07 (.474)
	No	100 (44.1)	97 (42.7)	197 (86.8)	
Intermenstrual bleeding	Yes	16 (7.0)	31 (13.7)	47 (20.7)	6.90 (.009)
	No	100 (44.1)	80 (35.2)	180 (79.3)	
Menorrhagia	Yes	13 (5.7)	42 (10.1)	58 (15.8)	21.91 (<.001)
	No	103 (45.4)	69 (38.8)	172 (84.2)	
Worsening of premenopausal syndrome	Yes	23 (10.1)	36 (15.9)	59 (26.0)	4.66 (.030)
	No	93 (41.0)	75 (33.0)	168 (74.0)	

COVID-19: Coronavirus disease 2019.

**Table 3.** Vaccine type and management in women experiencing menstrual abnormalities after COVID-19 vaccination (N=227)

Characteristic	Label (range)	Post-vaccination menstrual cycle changes, n (%)		
		No (n=116)	Yes (n=111)	Total
Dose after which symptoms appeared^†^	1st dose	34 (56.8)	55 (43.2)	89 (25.9)
	2nd doses	21 (6.1)	52 (15.2)	73 (21.3)
	3rd doses	35 (10.2)	52 (15.2)	87 (25.4)
	4th doses	33 (9.6)	61 (17.8)	94 (27.4)
Type of vaccine administered^†^	Pfizer-BioNTech	213 (23.5)	186 (20.5)	399 (43.9)
	Moderna	41 (4.5)	37 (4.1)	78 (8.6)
	AstraZeneca	38 (4.2)	36 (4.0)	74 (8.1)
	Sinopharm	16 (1.8)	31 (3.4)	47 (5.2)
	Novavax	0 (0)	3 (0.4)	2 (0.9)
	Johnson & Johnson	1 (0.1)	1 (0.3)	1 (0.4)
Management of post-vaccination menstrual abnormalities^†^	Consulted relatives or acquaintances	62 (37.8)	22 (13.4)	84 (51.2)
	Searched symptoms online	10 (6.1)	25 (15.2)	35 (21.3)
	Consulted primary care physician	4 (2.4)	20 (12.2)	24 (14.6)
	Self-treatment	3 (1.8)	14 (8.5)	17 (10.4)
	Received gynecological medical care	0 (0)	4 (2.4)	4 (2.4)

COVID-19: Coronavirus disease 2019.

† Multiple response.

**Table 4.** Multivariate logistic regression analysis of factors influencing menstrual cycle changes following COVID-19 vaccination (N=227)

Characteristics	Label (range)	Odds ratio (95% CI)	*p*
Age (year)	15–19	0.25 (0.01–4.36)	.006
	20–29	0.13 (0.04–0.41)	.343
	30–39	0.33 (0.11–1.03)	<.001
	40–49	Reference	
Body mass index (kg/m^2^)	<18.5 (underweight)	0.03 (0.01–0.10)	.030
	18.5–22.9 (normal)	0.002 (0.01–0.05)	.002
	23–24.9 (overweight)	0.18 (0.04–0.27)	.181
	>25 (obesity)	Reference	
Marital status	No	3.02 (1.24–7.38)	.015
	Yes	Reference	
Previous COVID-19 infection	No	3.46 (1.27–9.43)	.015
	Yes	Reference	
Number of COVID-19 vaccinations relating to menstrual abnormalities	Once or twice	Reference	
	Three times or more	2.07 (0.94–4.55)	.015
Intermenstrual bleeding	No	Reference	
	Yes	2.72 (1.06–6.96)	.037
Menorrhagia	No	Reference	
	Yes	5.37 (2.05–14.09)	<.001
Menstrual irregularities (day)	Regular (21–35)	Reference	
	Irregular (<21 or >35)	17.26 (6.51–45.75)	<.001

COVID-19: Coronavirus disease 2019; CI, confidence interval.
